# Clinical progression, pathological characteristics, and radiological findings in children with diffuse leptomeningeal glioneuronal tumors: A systematic review

**DOI:** 10.3389/fonc.2022.970076

**Published:** 2022-09-16

**Authors:** Haoxiang Jiang, Lu Qiu, Juan Song, Dandan Xu, Lei Sun, Yinbo Feng, Jun Zhao, Jun Qian, Zhiwei Yu, Jin Peng

**Affiliations:** ^1^ Department of Radiology, Wuxi Children’s Hospital Affiliated to Nanjing Medical University, Wuxi, China; ^2^ Department of Pediatrics, Wuxi Children’s Hospital Affiliated to Nanjing Medical University, Wuxi, China; ^3^ Department of Radiology, Xi’an Children’s Hospital, Xi’an, China

**Keywords:** clinical progression, pathology, radiology, Diffuse Leptomeningeal Glioneuronal Tumor, pediatrics, systematic review

## Abstract

**Background:**

Diffuse leptomeningeal glioneuronal tumors are rare leptomeningeal neoplasms composed of oligodendrocyte-like cells characterized by neuronal differentiation and a lack of isocitrate dehydrogenase gene mutation.

**Purpose:**

We aimed to analyze the clinical progression, pathological characteristics, and radiological findings of diffuse leptomeningeal glioneuronal tumors in children, as well as the relevance of clinico-radiological data.

**Data Sources:**

We searched MEDLINE, PubMed, and Web of Science to identify case reports, original articles, and review articles discussing diffuse leptomeningeal glioneuronal tumors published between 2000 and 2021.

**Study Selection:**

The analysis included 145 pediatric patients from 43 previous studies.

**Data Analysis:**

Data regarding patient pathology, MRI manifestations, clinical symptoms, and progression were collected. The relationship between imaging classification and pathological findings was using chi-square tests. Overall survival was analyzed using Kaplan–Meier curves.

**Data Synthesis:**

Parenchymal tumors were mainly located in the intramedullary areas of the cervical and thoracic spine, and patients which such tumors were prone to 1p-deletion (χ^2 =^ 4.77, p=0.03) and KIAA1549-BRAF fusion (χ^2 =^ 12.17, p<0.001). The median survival time was 173 months, and the survival curve fell significantly before 72 months. Parenchymal tumor location was associated with overall survival (p=0.03), patients with KIAA 1549-BRAF (+) and treated with chemotherapy exhibited a better clinical course (p<0.001).

**Limitations:**

The analysis included case reports rather than consecutively treated patients due to the rarity of diffuse leptomeningeal glioneuronal tumors, which may have introduced a bias.

**Conclusions:**

Early integration of clinical, pathological, and radiological findings is necessary for appropriate management of this tumor, as this may enable early treatment and improve prognosis.

## Introduction

The 2021 World Health Organization (WHO) classification of brain tumors lists diffuse leptomeningeal glioneuronal tumor (DLGNT) as a tumor type alone (1), which has not yet been assigned a WHO grade. DLGNTs are rare leptomeningeal neoplasms composed of oligodendrocyte-like cells characterized by neuronal differentiation and a lack of isocitrate dehydrogenase (IDH) gene mutation. Additionally, DLGNTs have been associated with KIAA1549-BRAF gene fusion and 1p deletion or 1p/19q co-deletion (2, 3). Given the rarity of this tumor type, its histological, radiological, and clinical features and pattern of progression remain to be fully elucidated (4).

Although they frequently occur as leptomeningeal tumors (5), some studies have reported DLGNTs without leptomeningeal dissemination, which manifests as intraspinal or intracerebral cystic or solid masses (6–8). Notably, clinical follow-up is often incomplete, and some patients present with indolent chronic disease while others experience an aggressive clinical course (4). Moreover, the differential diagnosis often includes tuberculous meningitis, cryptococcal neoformans meningitis, and meningeal metastatic tumor. Therefore, a systematic review may aid in clarifying the characteristics of DLGNT, which can in turn promote early treatment and improve prognosis/management.

DLGNT mainly occurs in children, and adult cases are rare. In this review, we focused on literature concerning pediatric DLGNT published from 2000 to 2021. To determine the relevance of clinico-radiological data for diagnosis and decision-making concerning DLGNT, we aimed to analyze the clinical progression, pathological characteristics, and MRI findings among these cases.

## Materials and methods

This study was conducted according to the Preferred Reporting Items for Systematic reviews and Meta-Analyses (9).

### Literature search

We searched for articles related to DLGNT, published between 2000 and 2021, using PubMed, MEDLINE, and Web of Science. The following keywords were used: diffuse leptomeningeal glioneuronal tumor, DLGNT, diffuse leptomeningeal oligodendroglioma, and diffuse leptomeningeal gliomatosis/neurocytomas. Inclusion criteria were as follows: (i) age ≤18 years; (ii) availability of MRI and clinical data; (iii) case reports, original articles, and review articles. Exclusion criteria were as follows: (i) incomplete MRI or clinical data; (ii) meeting abstracts, letters, or comments; (iii) lack of relevant data; (iv) radiology or pathology not conforming to the diagnostic criteria for DLGNT (10).

### Quality assessment and data extraction

Risk of bias and adequacy of reporting were assessed by two investigators using the Quality Assessment Tool for case reports published by the Australia Joanna Briggs Institute (https://synthesismanual.jbi.global) (11). For each case, the first author, publication year, country, age, sex, cerebrospinal fluid analysis, progression, pathology, and MRI manifestations were documented. All imaging studies were independently reviewed by two trained radiologists (LQ, with 3 years of pediatric radiology experience and JS, with 8 years of pediatric radiology experience). If no agreement could be reached, a decision was made in consultation with a third author (HJ, with 17 years of pediatric radiology experience).

### Statistical analysis

Clinical data were described using ranges, mean value, medians, and percentages as appropriate. The relationships between imaging classifications and pathological findings were analyzed using chi-square tests. The difference in prognosis between surgical resection and nonsurgical resection groups were analyzed using chi-square tests. Overall survival was analyzed using Kaplan–Meier curves and the log-rank test. P values <0.05 were considered significant.

## Results

### Study selection

**Figure 1:**
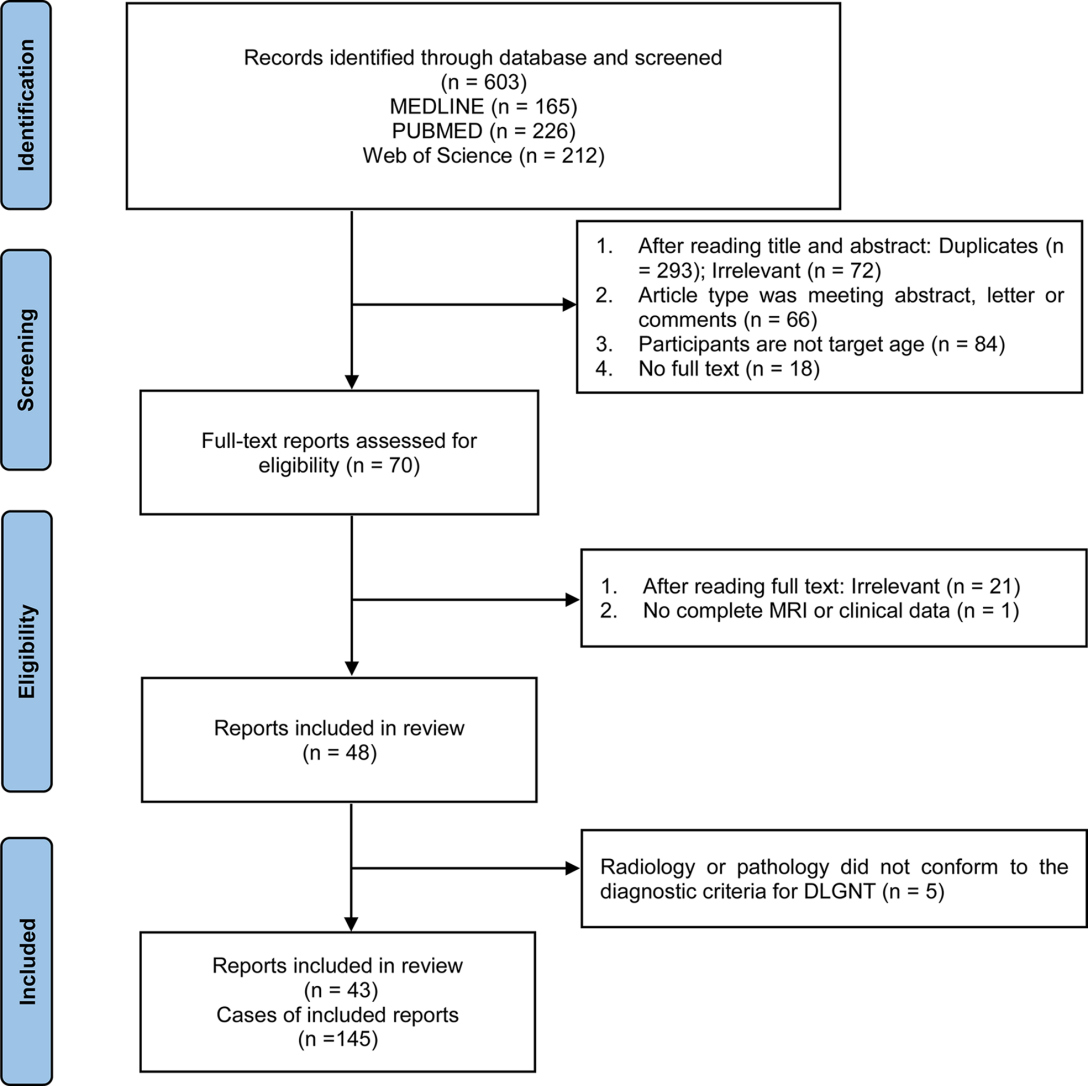
Flow chart showing the selection of reports.

**Figure 1** displays the flow of the review process. The initial search of electronic databases identified 603 records. The titles and abstracts of these records were screened for their eligibility, resulting in a first selection of 70 papers. The full text was further assessed for each of these articles. Studies were excluded if they were irrelevant to our purpose (n = 21) or did not include complete MRI or clinical data (n = 1). Among the 48 studies remaining, 43 studies were finally included based on the diagnostic criteria for DLGNT. The analyses thus included 145 patients from the previous literature. Based on the risk of bias and adequate reporting assessment, studies were rated as good (**Online Tables 1-2**).

### Clinical data

**Figure 2:**
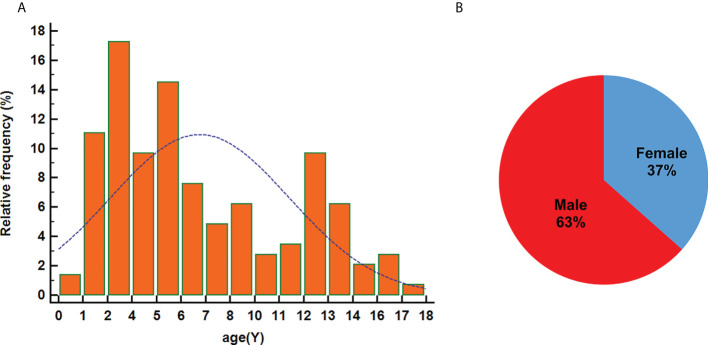
Age at diagnosis **(A)** and sex distribution **(B)**.

In the combined analysis of the previous literature [145 pediatric patients with DLGNT (**Table 1** and **Figure 2**); average age: 6.91 years; median age: 5 years], male predominance was observed in the overall sample (male/female: 1.7/1). The most reported symptoms were headache, nausea, and vomiting, which may have been related to hydrocephalus. Other symptoms included seizures, ataxia, speech problems, and sensorimotor impairments. Cerebrospinal fluid samples were positive for protein and negative for malignant cells except 3 cases, of which 2 cases showed malignant tumor cells with increased protein content and 1 case showed normal protein content without malignant tumor cells.

**Table 1:** Literature review of DLGNT.

Author	Date	Country	Samplesize	Age (year)	Gender(M/F)	Cerebrospinal fluid	Treatment	Statement	AWD (months)	DOD (months)
Armao (12)	2000	America	1	8	M	Pro (+);MTC (-)	/	Dead		60
Perilongo (13)	2002	Italy	3	3.33-12	2M/1F	Pro (+);MTC (-)	RT; 2 CHT	2Dead; 1Stable	24	9-108
Stödberg (14)	2002	Sweden	1	2	M	NA	CHT	Stable	23	
Bourne (15)	2006	America	1	2	M	Pro (+);MTC (-)	CHT	Stable	16	
King (16)	2008	Canada	1	12	M	Pro (+);MTC (+)	ECT+CHT	DET	8	
Gardiman (17)	2010	Italy	4	3-13	2M/2F	Pro (+);MTC (-)	CHT; ECT+CHT; ECT+CHT+RT; /	2Stable; 1Dead; 1DET	18-72	72
Demir (18)	2010	Turkey	1	8	F	Pro (+)	ECT+CHT+RT	Stable	19	
Hervey-Jumper (19)	2010	America	1	9	F	Pro (+);MTC (-)	NA	NA	NA	
Agamanolis (20)	2012	America	3	4-9	2M/1F	Pro (+);MTC (-)	CHT; ECT; CHT+RT	2Stable; 1Dead	36-48	24
Rodriguez (2)	2012	America	33	0.5-16	22M/11F	Pro(+);MTC(-)	3ECT; 20CHT; 7RT	9Stable; 2DET; 8Dead		2-72
Schniederjan (21)	2013	America	9	1.5-7	4M/5F	MTC (-)	4CHT; 1CHT+RT; 1RT	4Stable; 3DET	24-137	
Cho (22)	2014	Korea	1	11	M	NA	CHT	DET	23	
Kosker (23)	2014	Turkey	1	3	M	Pro (+);MTC (-)	CHT	Stable	18	
Lee (24)	2014	America	1	15	M	Pro (+);MTC (-)	ECT+CHT+RT	Stable	104	
Kessler (25)	2015	America	1	12	F	Pro (+);MTC (-)	CHT	Dead		13
Preuss (26)	2015	Germany	4	1.9-8.75	4M	Pro (+);MTC (-)	3CHT; 1CHT+RT	3Stable; 1Dead	40-96	19
Lyle (27)	2015	America	1	14	F	Pro (+);MTC (-)	CHT+RT	Stable	25	
Chellathurai (28)	2016	India	1	2	M	Pro (+);MTC (-)	Intensive Treatment	Dead		4
Dodgshun (29)	2016	America	10	1.59-14.08	6M/4F	NA	8CHT; 1CHT+RT	8Stable; 2Dead	6-69	60-69
Dyson (30)	2016	America	1	15	M	NA	ECT+CHT+RT	DET	3	
GuillénQuesada (31)	2017	Spain	1	13	F	Pro (+);MTC (-)	CHT	Dead		7
Aguilera (32)	2017	America	7	2-7	4M/3F	MTC (-)	7CHT	7Stable	15-164	
Chiang (6)	2017	America	4	5-14	3M/1F	NA	NA	4Stable	2-90	
Karlowee (33)	2017	Japan	1	17	M	Pro (+);MTC (-)	CHT+RT	DET	10	
Schwetye (34)	2017	America	2	7-9	2M	Pro (+);MTC(+); Pro(-);MTC (-)	CHT+RT; ECT+CHT	2DET	12 -36	
Nambirajan (35)	2018	India	1	13	F	Pro (+)	ECT	Dead		4
Deng (36)	2018	Germany	24	2-14	12M/12F	NA	Extensive treatment	8Stable; 3DET; 4Dead	2-286	48-173
Tan (37)	2019	Singapore	1	4	M	Pro (+);MTC (-)	ECT+CHT	Stable	NA	
Kurozumi (38)	2019	Japan	1	13	F	NA	ECT+CHT	DET	18	
Qian (39)	2019	China	1	2	NA	Pro (+)	ECT	Dead		3
Deng (36)	2019	China	1	9	M	Pro (+);MTC (-)	ECT	NA	NA	
Tiwari (40)	2019	America	1	13	F	NA	CHT	Stable	18	
Tiwari (41)	2019	India	1	3	F	Pro (+)	CHT	NA	NA	
Bao (42)	2019	China	1	16	F	Pro (+)	NA	NA	NA	
Abongwa (43)	2020	America	3	2.5-6	2M/1F	NA	2CHT 1CHT+RT	2DET; 1Dead	156-204	12
Lakhani (44)	2020	America	7	3-14	7 M	NA	NA	NA	NA	
Sáez-Alegre (45)	2020	Spain	1	3	M	MTC (-)	ECT+CHT	Stable	5	
SiqinZhou (46)	2020	China	1	16	F	Pro(+),MTC (-);	Expectant treatment	Stable	NA	
Valiakhmetova (47)	2020	Russia	2	2.25-8	1M/1F	NA	1CHT; 1Targeted therapy	2Stable	24-25	
Chen (48)	2020	China	1	12	M	MTC (-)	CHT	Dead		16
Manoharan (4)	2021	Australia	2	8-13	1M/1F	MTC (-)	2CHT	1Stable; 1DET	6-16	
Karimzadeh (49)	2021	Iran	1	10	M	Pro (+); MTC (-)	CHT	Stable	22	
Teh (50)	2021	Malaysia	1	5	M	Pro (+); MTC (-)	Palliative treatment	Dead		5

Pro, protein; MTC, malignant cells; NA, inability to perform; RT, radiotherapy; ECT, ectomy; CHT, chemotherapy; DET, deteriorate AWD, alive with disease; DOD, death of disease.

### Pathological findings

**Figure 3:**
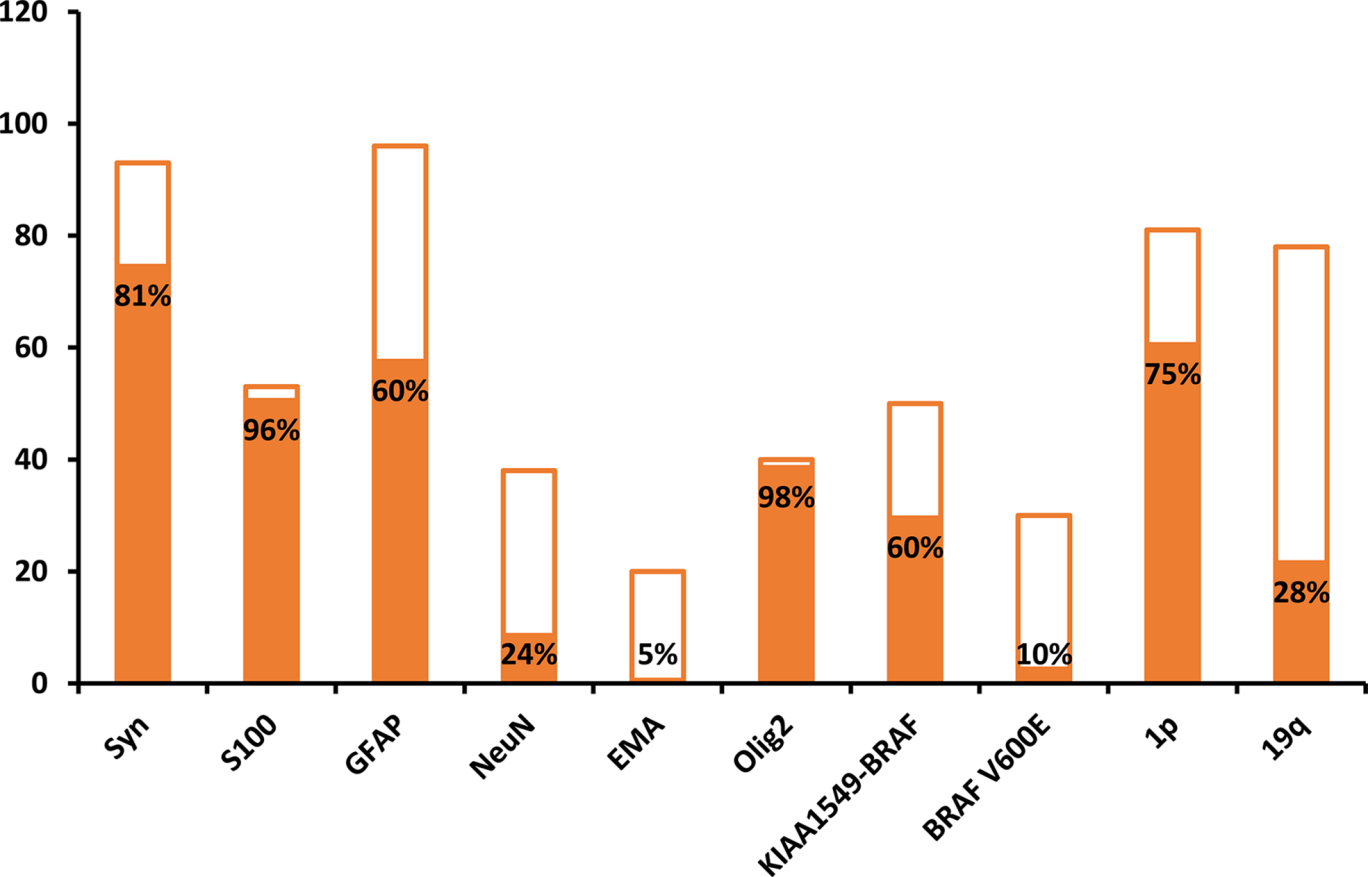
Analysis of immunohistochemical and molecular markers in patients with diffuse leptomeningeal glioneuronal tumors. Y-axis stands for the number of cases, and the solid bars represent the percentage of positive cases.

The combined analysis of data from previously published cases revealed that the common histological findings were oligodendrocyte-like cells in the desmoplastic leptomeninges, which were composed of round to ovoid nuclei with mild atypia, clear cytoplasm, and perinuclear halos. Most DLGNTs were histologically low-grade, with a Ki67 index of <20%. Among patients with available data (**Figure 3**) (**Online Table 3**), S100 and oligodendrocyte transcription factor 2 positivity were observed in 96% (51 of 53) and 98% (39 of 40) of cases, respectively. All patients with available data were negative for IDH1 (0 of 53), while 60% (58 of 96) were positive for glial fibrillary acidic protein positive, and 81% (75 of 93) were positive for synaptophysin. In addition, neuronal nuclear antigen findings were positive in 24% (9/38) of patients, while epithelial membrane antigen findings were positive in only 4% (1/20) of patients. Moreover, 60% (30/50) of patients exhibited KIAA1549-BRAF fusion, 10% (3/30) exhibited BRAF V600E mutation, 75% (61/81) exhibited 1p-deletion, 28% (22/78) exhibited 19q-deletion, and 27% (21/78) exhibited co-deletion of 1p/19q.

### Radiological findings

**Figure 4:**
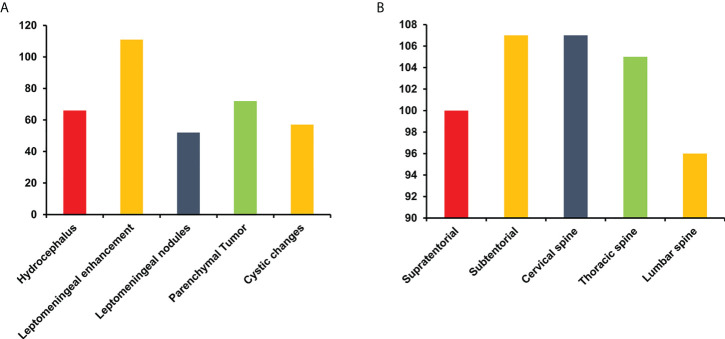
The finding **(A)** and distribution of lesions **(B)** on MRI in patients with diffuse leptomeningeal glioneuronal tumors. Y-axis represents the number of cases.

**Figure 5:**
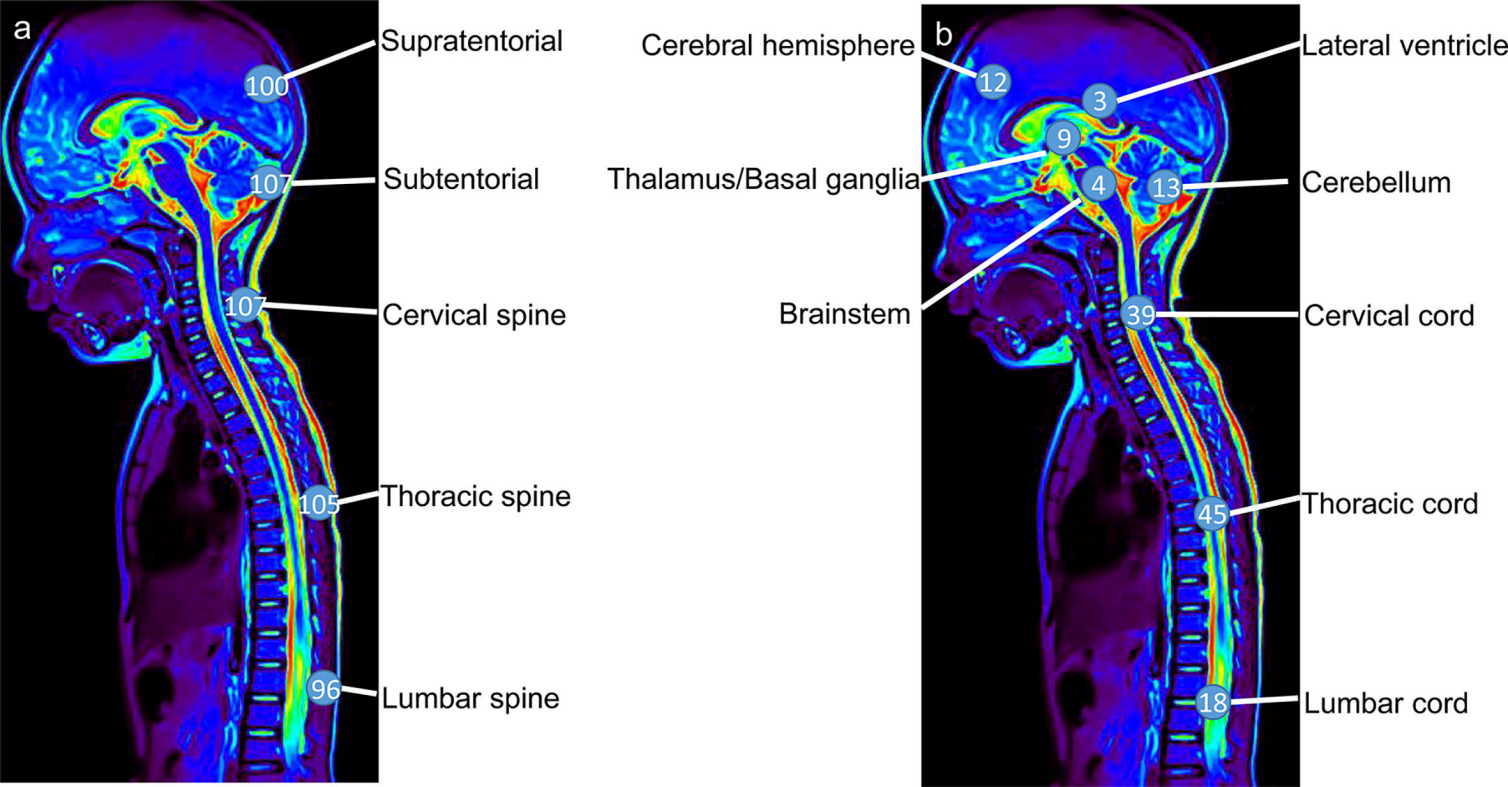
The lesions **(A)** and parenchymal tumor **(B)** location in patients with diffuse leptomeningeal glioneuronal tumors. Numbers in circles represent the numbers of lesions **(A)** and parenchymal tumors **(B)** in the specific region.

Sixty-nine percent (66/95) of patients with available data presented with hydrocephalus, which was the most common finding at the first hospital admission and was associated with headache and vomiting. In addition, 85% (111/131) of patients developed extensive contrast enhancement of the intracranial and spinal leptomeninges on MRI. Nodular and cystic changes of the leptomeninges were also detected in 50% (52/103) and 52% (57/109) of patients, respectively. Unusual unequivocal masses and parenchymal invasion were observed in 53% (72/137) of patients (**Figure 4A**). DLGNT lesions were mainly located in the subtentorial area (72%) (107/148), cervical spinal (72%) (107/148) and thoracic spine (71%) (105/148) (**Figures 4B**, **5A**). In contrast, parenchymal tumors were mainly located in the intramedullary regions of the cervical (53%) (39/73) and thoracic spine (62%) (45/73) (**Figure 5B**).

In addition, patients with parenchymal tumors were prone to 1p-deletion (χ^2 =^ 4.77, p=0.03) and KIAA1549-BRAF fusion (χ^2 =^ 12.17, p<0.001). Other imaging classifications were not significantly associated with pathological findings (p>0.05).

### Treatments and prognosis

In total 145 cases, 94 patients were successfully followed up for 2–286 months (median follow-up: 25 months). 70% (66/94) of follow-up patients were still living. The total mortality rate was 19% (28/145). Among the 28 deceased patients, only 7 underwent postmortem examinations. One patient died of sepsis and multiple organ failure, and an autopsy revealed extensive necrotizing pneumonia due to *Legionella pneumophila*. More patients died after a devastating course of progressive neurological deterioration. Of the 20 patients who underwent surgical tumor resection, 18 were successfully followed up, of whom 5 died, 6 progressed and 7 were stable. Average survival time was 36 months with a 25% (5/20) overall mortality rate. Of the 74 patients who underwent biopsy due to the multifocality of lesions, 56 were successfully followed up, of whom 16 died, 10 progressed and 40 were stable. Average survival was 45 months with a 22% (16/74) overall mortality rate. There was no significant difference in prognosis between tumor resection and non-resection groups (χ2 = 5.00, P=0.08). Chemotherapy (85%) (79/93) was usually performed following confirmation of the diagnosis. Radiotherapy and ventricular-peritoneal shunting were performed in 23% (21/93) and 38% (36/94) of patients, respectively.

Furthermore, different molecular subtypes have different prognoses. 23 KIAA1549-BRAF positive patients were followed up, of which 5 died, 4 progressed and 14 stable. All 3 BRAF-V600E positive patients were stable. 42 1p deleted patients were followed up, of which 8 died, 9 progressed, and 25 stable. 14 19q deleted patients were followed up, of which 3 died, 3 progressed and 8 stable.

**Figure 6:**
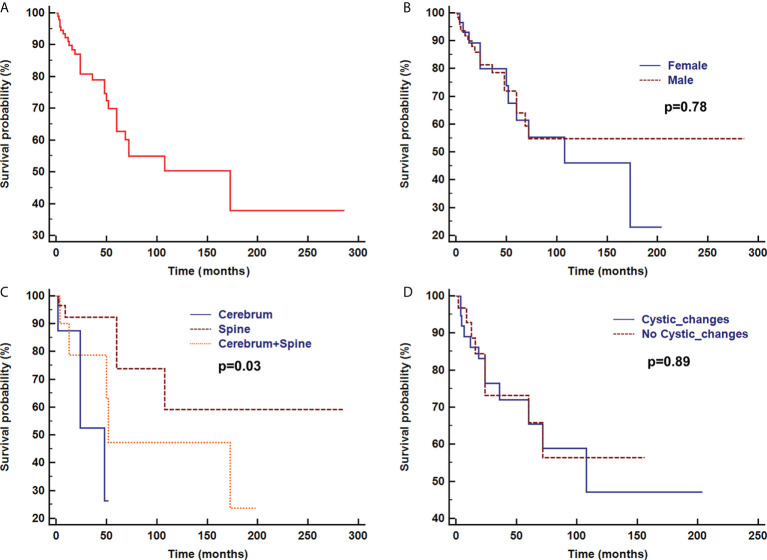
Survival curve analysis of overall survival **(A)**, sex **(B)**, and radiological findings of parenchymal tumor location **(C)**, and with or without cystic changes **(D)** were analyzed using Kaplan–Meier curves and log-rank tests.

**Figure 7:**
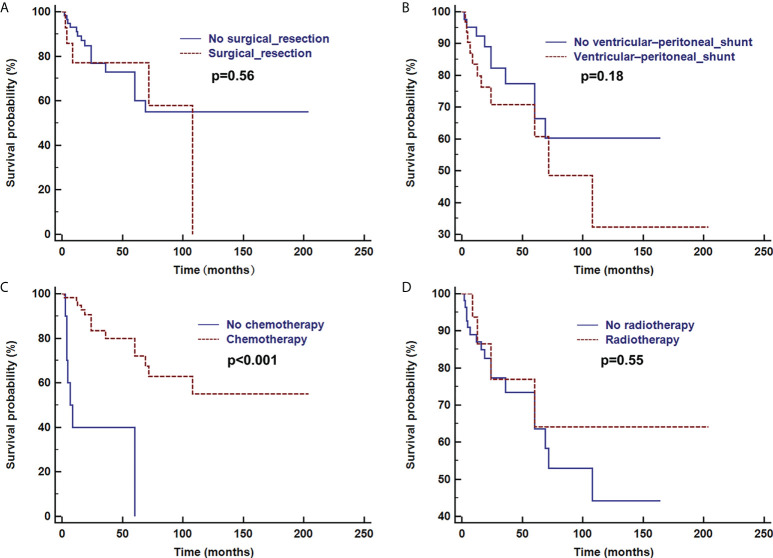
Survival curve analysis of treatment with or without surgical resection **(A)**, ventricular-peritoneal shunt **(B)**, chemotherapy **(C)**, and radiotherapy **(D)** were analyzed using Kaplan–Meier curves and log-rank tests.

**Figure 8:**
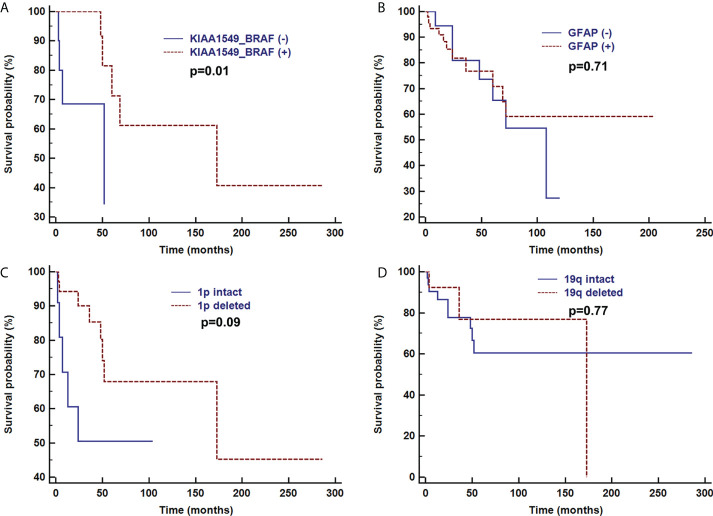
Survival curve analysis of pathological findings of KIAA1549-BRAF **(A)**, GFAP **(B)**, 1p intact **(C)**, and 19q intact **(D)** were analyzed using Kaplan–Meier curves and log-rank tests.

Overall survival time was analyzed after the first pathologic diagnosis (**Figure 6**). The median survival time was 173 months, and the survival curve fell significantly before 72 months. For patients with parenchymal tumors, location was associated with overall survival (60 months; 26% for those located in the cerebrum vs. 74% located in spine, p=0.03). Patients treated with chemotherapy exhibited a better clinical course (overall survival of 60 months, 0% vs. 72% with chemotherapy, p<0.001) (**Figure 7**). In immunohistochemical, KIAA-BRAF positive patients had a better clinical course than negative patients (overall survival of 60 months, 34% vs. 71%, p=0.01). There were no differences in GFAP, 1p, 19q (**Figure 8**), or treatment with surgical resection, radiotherapy, or ventricular-peritoneal shunting (p>0.05).

## Discussion

In this study, we comprehensively reviewed DLGNT studies published from 2000 to 2021, following which we analyzed data for 145 pediatric patients included in 43 previous studies. To the best of our knowledge, this clinical imaging-focused series is the largest in the literature to date.

DLGNT is more common in children than in people aged >18 years and demonstrates a male predominance (2, 27). In this study, the peak age at onset ranged from 1–6 years old, with DLGNT affecting more boys than girls. In addition, 69% patients presented with hydrocephalus, which was associated with headache and vomiting caused by intracranial hypertension. It is a bite higher than previous literatures (2, 29). Some cases did not mention whether there was hydrocephalus. We did not take this part of cases into consideration, which may lead to literature bias.

The cerebrospinal fluid samples from most patients except 3 cases exhibit increased protein content without increases in cytological components, suggesting an increased likelihood of leptomeningeal proliferation of tumor cells (2). Two cases showed malignant tumor cells. For the first case, surgical resection and followed chemotherapy did not stop progression. The second patient died one year after diagnosis despite prompt chemotherapy. We cannot figure out whether there is a difference in prognosis between malignant tumor cells positive and negative patients due to the small sample size, further studies are needed in the future.

In total, 96% of patients tested were positive for S100 and negative for epithelial membrane antigen, indicating that the tumors originated from nerve cells rather than epithelial cells. Unlike gangliogliomas, DLGNTs are composed of a single-cell-type population of both glial and neuronal cell components. Consistently, 98% of patients tested positive for oligodendrocyte transcription factor 2, a transcription factor known as a common precursor of glial and neuronal cells (51). In our study, all patients with available data were negative for IDH1, consistent with previous findings (21).

In molecular testing, BRAF and 1p/19q gene alterations are important for differential diagnosis (3). Both pilomyxoid astrocytoma and DLGNT exhibit a tendency for leptomeningeal spread as well as similarities in histological features. However, pilomyxoid astrocytoma exhibits a mucinous background, in which bipolar tumor cells radiate around blood vessels along with rare gangliocytic tumor cells, and 1p/19q chromosome arm deletion is absent in such cases (2). A previous study reported 1p deletion and BRAF-KIAA1549 fusion in 59% and 75% of patients with DLGNT, respectively (3). Similarly, these mutations occurred in 75% and 60% of patients in this study. KIAA1549-BRAF fusion has also been associated with pilocytic astrocytoma (7). Thus, 1p deletion may represent a specific diagnostic index.

As noted in a previous MRI study (44), DLGNT presented with hydrocephalus, diffuse meningeal enhancement, nodules and diffuse cystic changes. The nodules and cystic changes mainly located the leptomeningeal surfaces of the brain and spine, as well as subependymal regions of the ventricle. A DLGNT autopsy study conducted by Louis (10) revealed extensive dilation and fibrosis of the subarachnoid space, accompanying intraventricular mass and cystic changes, as well as intraparenchymal extensions along the perivascular space. In this study, 53% of patients had parenchymal tumors in the cerebrum or spinal cord, which were mainly located in the intramedullary area of the cervical-thoracic cord. However, Chiang et al. (6) reported masses in the spinal cord but no diffuse lesions in their patients. This finding suggests the biological evolution of the tumor from the parenchyma to the cerebrospinal fluid and blood vessel space. Additionally, our findings indicated that patients with parenchymal tumors were prone to 1p-deletion and KIAA1549-BRAF fusion, highlighting the need for additional studies. Previous literature showed different molecular subtypes were associated with MRI findings that leptomeninges enhancement was more common in DLGNT-MC-2. (**Online Figure 1**) (7).

The biological behavior of DLGNT remains uncertain and has not been recommended by WHO for histological classification. Our study found most patients had stable prognosis. The 19% mortality rate supports the classification of DLGNT as an indolent disease in children. In this study, good clinical relief was observed after urgent ventriculoperitoneal shunting, although hyperproteinorrachia is often detected in these lesions, which can produce shunt obstruction (52). Surgical tumor resection and ventricular-peritoneal shunting alone are insufficient for improving overall survival, chemotherapy can significantly improve survival, as noted in previous studys (2, 53). Additionally, inhibition of the MAPK signaling pathway represents a potential therapeutic approach (29). In particular, patients with parenchymal tumors located in the cerebrum and those treated without chemotherapy experienced a worse clinical course, which suggests the importance of early diagnosis, repeated biopsy, localization, and standardized treatment.

In addition, previous research indicated histological and molecular features are associated with overall survival (2, 7). In our study, KIAA1549-BRAF positive patients had significantly higher overall survival than negative ones. However, there were no differences in GFAP, 1p, 19q.

Our study had some limitations, including its retrospective nature. In addition, the analysis included case reports rather than consecutively treated patients due to the rarity of DLGNT, which may have biased the results. Further, details regarding clinical progression and the extent of the correlation analysis between radiographic and histopathological features were limited to insufficient availability of follow-up data.

In conclusion, the current study suggests that DLGNT presents with specific, regular changes, including hydrocephalus, diffuse enhancement of the meninges, multiple nodules, and cystic changes on the brain surface. Notably, clinical progression, pathological characteristics, and radiological findings were associated with overall survival. Our findings highlight the need to integrate clinical and imaging features to improve the early and accurate diagnosis of DLGNT, simplify clinical decision-making, and improve prognosis.

## Data availability statement

The original contributions presented in the study are included in the article/**Supplementary Material**. Further inquiries can be directed to the corresponding authors.

## Author contributions

HJ and LQ drafted the initial manuscript, and summarized and analyzed the data. JS, DX, LS, YF, JZ, and JQ designed the data collection instruments, collected data, and carried out the initial analyses. JP and ZY conceptualized and designed the article, reviewed and revised the manuscript, and reviewed the manuscript for important intellectual content. All authors approved the final manuscript as submitted and agree to be accountable for all aspects of the work.

## Funding

Natural Science Foundation of Shaanxi Province (2021JM-558), Medical Innovation Team of Jiangsu Province (CXTDB2017016), and Wuxi Health Commission Precision Medicine Key Projects and Funding(j202107).

## Conflict of interest

The authors declare that the research was conducted in the absence of any commercial or financial relationships that could be construed as a potential conflict of interest.

## Publisher’s note

All claims expressed in this article are solely those of the authors and do not necessarily represent those of their affiliated organizations, or those of the publisher, the editors and the reviewers. Any product that may be evaluated in this article, or claim that may be made by its manufacturer, is not guaranteed or endorsed by the publisher.

## Supplementary material

Supplementary Figure 1 Radiological features of 3 representative cases of DLGNTs. [DLGNT-MC-1_10 **(A-C)**, DLGNT-MC-2_09 **(D, E)**, DLGNT-MC-2_03 **(F)**]. Multicystic lesions with nodular enhancement in the cerebellum and cervical spinal cord spanning with leptomeningeal enhancement covering the cerebellum. Reproduced with permission from Springer Nature. Deng MY, Sill M, Chiang J, et al. Molecularly defined diffuse leptomeningeal glioneuronal tumor (DLGNT) comprises two subgroups with distinct clinical and genetic features. Acta Neuropathol 2018;136(2):239-53.

## Abbreviations

DLGNT, diffuse leptomeningeal glioneuronal tumors; IDH, isocitrate dehydrogenase; WHO, World Health Organization.
