# Supplementary material for: Clinical progression, pathological characteristics, and radiological findings in children with diffuse leptomeningeal glioneuronal tumors: A systematic review
Source: Front Oncol. 2022 Sep 16;12:970076. doi: 10.3389/fonc.2022.970076 (PMC9525023; doi:10.3389/fonc.2022.970076)
Supplement: Supplementary file 2 [file DataSheet_1.docx]

Online Table 1 Quality Assessment Checklist by Australia Joanna Briggs Institute

| Items | Assessment | | | |
| --- | --- | --- | --- | --- |
|  | Yes | No | Unclear | Inapplicability |
| Is the review question clearly and explicitly stated? | √ |  |  |  |
| Were the inclusion criteria appropriate for the review question? | √ |  |  |  |
| Was the search strategy appropriate? | √ |  |  |  |
| Were the sources and resources used to search for studies adequate? | √ |  |  |  |
| Were the criteria for appraising studies appropriate? | √ |  |  |  |
| Was critical appraisal conducted by two or more reviewers independently? | √ |  |  |  |
| Were the methods used to combine studies appropriate? | √ |  |  |  |
| Was the likelihood of publication bias assessed? | √ |  |  |  |
| Were recommendations for policy and/or practice supported by the reported data? | √ |  |  |  |
| Were the specific directives for new research appropriate? | √ |  |  |  |

Online Table 2 Joanna Briggs Institute Critical Appraisal Checklist for Case reports

| Author | Q1 | Q2 | Q3 | Q4 | Q5 | Q6 | Q7 | Q8 |
| --- | --- | --- | --- | --- | --- | --- | --- | --- |
| Armao^19^ | Y | Y | Y | Y | N | Y | U | Y |
| Perilongo^20^ | Y | Y | Y | Y | Y | Y | Y | Y |
| Stödberg^21^ | Y | Y | Y | Y | Y | Y | U | Y |
| Bourne^22^ | Y | Y | Y | Y | Y | Y | Y | Y |
| King^23^ | Y | Y | Y | Y | Y | Y | U | Y |
| Gardiman^24^ | Y | Y | Y | Y | Y | Y | U | Y |
| Demir^25^ | Y | Y | Y | Y | Y | Y | U | Y |
| Hervey-Jumper^26^ | Y | Y | U | Y | U | N | U | Y |
| Agamanolis^27^ | Y | Y | Y | Y | Y | Y | U | Y |
| Rodriguez^2^ | Y | N | Y | Y | Y | Y | U | Y |
| Schniederjan^14^ | Y | N | Y | Y | Y | Y | U | Y |
| Cho^28^ | Y | Y | Y | Y | Y | Y | Y | Y |
| Kosker^29^ | Y | Y | Y | Y | Y | Y | U | Y |
| Lee^30^ | Y | Y | Y | Y | Y | Y | U | Y |
| Kessler^31^ | Y | Y | Y | Y | Y | Y | U | Y |
| Preuss^32^ | Y | Y | Y | Y | Y | Y | Y | Y |
| Lyle^12^ | Y | Y | Y | Y | Y | Y | U | Y |
| Chellathurai^33^ | Y | Y | Y | Y | Y | Y | U | Y |
| Dodgshun^18^ | Y | N | Y | Y | Y | Y | U | Y |
| Dyson^34^ | Y | Y | Y | Y | Y | Y | U | Y |
| GuillénQuesada^35^ | Y | Y | Y | Y | Y | Y | U | Y |
| Aguilera^36^ | Y | N | Y | Y | Y | Y | U | Y |
| Chiang^6^ | Y | N | Y | Y | U | Y | U | Y |
| Karlowee^37^ | Y | Y | Y | Y | Y | Y | U | Y |
| Schwetye^38^ | Y | Y | Y | Y | Y | Y | U | Y |
| Nambirajan^39^ | Y | Y | Y | Y | Y | Y | U | Y |
| Deng^40^ | Y | N | Y | Y | Y | Y | U | Y |
| Tan^41^ | Y | N | Y | Y | Y | Y | Y | Y |
| Kurozumi^42^ | Y | Y | Y | Y | Y | Y | Y | Y |
| Qian^43^ | Y | Y | Y | Y | Y | Y | Y | Y |
| Deng^40^ | Y | Y | U | Y | Y | N | U | Y |
| Tiwari^44^ | Y | Y | Y | Y | Y | Y | U | Y |
| Tiwari^45^ | Y | Y | U | Y | Y | N | U | Y |
| Bao^46^ | Y | Y | U | Y | U | N | U | Y |
| Abongwa^47^ | Y | Y | Y | Y | Y | Y | Y | Y |
| Lakhani^15^ | Y | N | U | Y | U | N | U | Y |
| Sáez-Alegre^48^ | Y | Y | Y | Y | Y | Y | U | Y |
| SiqinZhou^49^ | Y | Y | Y | Y | Y | Y | U | Y |
| Valiakhmetova^50^ | Y | Y | Y | Y | Y | Y | Y | Y |
| Chen^51^ | Y | Y | Y | Y | Y | Y | Y | Y |
| Manoharan^4^ | Y | Y | Y | Y | Y | Y | U | Y |
| Karimzadeh^52^ | Y | Y | Y | Y | Y | Y | Y | Y |
| Teh^53^ | Y | Y | Y | Y | Y | Y | U | Y |

Y: YES; N: No/Not applicable; U: Unclear; Q1:Were patient’s demographic characteristics clearly described? Q2:Was the patient’s history clearly described and presented as a timeline? Q3: Were valid methods used for identification of the condition for all participants included in the case series? Q4: Were diagnostic tests or assessment methods and the results clearly described? Q5: Was the intervention(s) or treatment procedure(s) clearly described? Q6: Was the post-intervention clinical condition clearly described? Q7: Were adverse events (harms) or unanticipated events identified and described? Q8: Does the case report provide takeaway lessons?

Online Table 3 Immunohistochemical of DLGNT

| Author | Sample date | Syn | S100 | GFAP | NeuN | EMA | Olig2 | KIAA1549-BRAF | BRAF-V600E | 1p | 19q |
| --- | --- | --- | --- | --- | --- | --- | --- | --- | --- | --- | --- |
| Armao^19^ | 1 | / | + | - | / | / | / | / | / | / | / |
| Perilongo^20^ | 3 | 1+;  2- | / | 1+;  2- | / | / | / | / | / | / | / |
| Stödberg^21^ | 1 | / | + | / | / | / | / | / | / | / | / |
| Bourne^22^ | 1 | - | + | - | - | - | / | / | / | + | - |
| King^23^ | 1 | / | + | + | / | / | / | / | / | / | / |
| Gardiman^24^ | 4 | 4+ | 4+ | 4+ | 1+;  3/ | 4- | / | / | / | 1+;  3/ | 1-;  3/ |
| Demir^25^ | 1 | / | / | + | / | / | / | / | / | / | / |
| Hervey-Jumper^26^ | 1 | + | / | / | / | / | / | / | / | + | - |
| Agamanolis^27^ | 3 | 3- | 3+ | / | / | / | / | / | / | 2+;1- | 3- |
| Rodriguez^2^ | 33 | 19;  8-;  6/ | 11+;  1-;  21/ | 12+;  19-;  2/ | 14-;19/ | 10-;  23/ | 9+;  24/ | / | / | 11+;  4-;  18/ | 3+;  12-;  18/ |
| Schniederjan^14^ | 9 | 8+;  1- | 9+ | 4+;  5- | 6-;  3/ | / | / | / | / | 6+;  2-;  1/ | 2+;  5-;  2/ |
| Cho^28^ | 1 | + | / | + | + | / | + | / | / | / | / |
| Kosker^29^ | 1 | / | + | / | / | / | + | / | / | / | / |
| Lee^30^ | 1 | + | / | + | - | / | / | / | / | - | - |
| Kessler^31^ | 1 | + | / | / | - | / | + | / | / | - | - |
| Preuss^32^ | 4 | 3-;  1+ | 3+;  1/ | 2+;  2- | / | / | 3+;  1/ | / | / | 2-;  2/ | 2-;  2/ |
| Lyle^12^ | 1 | + | / | + | / | / | + | / | / | / | / |
| Chellathurai^33^ | 1 | / | / | / | / | / | / | / | / | - | - |
| Dodgshun^18^ | 10 | 10+ | / | 10+ | / | / | 10+ | 4+;  2-;  4/ | 1+;  4-;  5/ | / | / |
| Dyson^34^ | 1 | + | / | + | - | / | / | / | / | - | - |
| GuillénQuesada^35^ | 1 | / | / | / | / | / | / | - | - | - | / |
| Aguilera^36^ | 7 | 7+ | 7+ | 7+ | / | / | / | / | 7- | / | / |
| Chiang^6^ | 4 | 4+ | / | 4- | 4+ | / | 4+ | 3+;  1/ | 3-;  1/ | 4+ | 3+;  1/ |
| Karlowee^37^ | 1 | + | + | + | / | / | + | / | / | + | - |
| Schwetye^38^ | 2 | 2+ | 1+;  1- | 1+;  1- | 2- | 1+;  1- | 1+;  1- | 2- | 2- | 1+;  1- | 2- |
| Nambirajan^39^ | 1 | + | / | + | + | - | / | - | - | + | + |
| Deng^40^ | 24 | / | / | / | / | / | / | 17+;  7- | 6-;  18/ | 24+ | 8+;  16- |
| Tan^41^ | 1 | + | + | + | / | / | / | + | / | + | - |
| Kurozumi^42^ | 1 | + | / | - | - | / | + | - | - | + | + |
| Qian^43^ | 1 | + | + | + | - | - | + | - | - | + | - |
| Deng^40^ | 1 | + | / | + | + | / | - | / | - | + | - |
| Tiwari^44^ | 1 | / | / | - | / | / | + | / | / | / | / |
| Tiwari^45^ | 1 | / | / | / | / | / | + | / | / | / | / |
| Bao^46^ | 1 | / | / | / | / | / | / | / | / | / | / |
| Abongwa^47^ | 3 | 3+ | 2+;  1/ | 3+ | / | / | / | / | / | / | / |
| Lakhani^15^ | 7 | / | / | / | / | / | / | 2+;  5- | / | 1+;  6- | 2+;  5- |
| Sáez-Alegre^48^ | 1 | + | / | + | / | / | / | / | + | + | + |
| SiqinZhou^49^ | 1 | / | / | / | / | / | / | / | / | / | / |
| Valiakhmetova^50^ | 2 | / | / | / | / | / | / | 1+;  1/ | 1+;  1/ | / | / |
| Chen^51^ | 1 | + | + | + | - | - | + | / | / | / | / |
| Manoharan^4^ | 2 | / | / | 1+;  1/ | 1+;  1/ | / | 1+;  1/ | 2+ | 2/ | 2+ | 1+;  1/ |
| Karimzadeh^52^ | 1 | + | + | - | / | - | + | / | / | / | / |
| Teh^53^ | 1 | + | + | / | / | / | / | / | / | / | / |
